# Reporter-Phage-Based Detection and Antibiotic Susceptibility Testing of *Yersinia pestis* for a Rapid Plague Outbreak Response

**DOI:** 10.3390/microorganisms9061278

**Published:** 2021-06-11

**Authors:** Sarit Moses, Moshe Aftalion, Emanuelle Mamroud, Shahar Rotem, Ida Steinberger-Levy

**Affiliations:** Department of Biochemistry and Molecular Genetics, The Israel Institute for Biological Research, Ness-Ziona 74100, Israel; Sarit5761@gmail.com (S.M.); moshea@iibr.gov.il (M.A.); emmym@iibr.gov.il (E.M.); Shaharr@iibr.gov.il (S.R.)

**Keywords:** reporter bacteriophage, rapid antibiotic susceptibility testing, antibiotic resistance, *Yersinia pestis*, plague, outbreak, clinical sample, environmental sample

## Abstract

Pneumonic plague is a lethal infectious disease caused by *Yersinia pestis*, a Tier-1 biothreat agent. Antibiotic treatment can save infected patients; however, therapy should begin within 24 h of symptom onset. As some *Y. pestis* strains showed an antibiotic resistance phenotype, an antibiotic susceptibility test (AST) must be performed. Performing the Clinical and Laboratory Standards Institute (CLSI)-recommended standard process, which includes bacterial isolation, enumeration and microdilution testing, lasts several days. Thus, rapid AST must be developed. As previously published, the *Y. pestis*-specific reporter phage ϕA1122::*luxAB* can serve for rapid identification and AST (ID-AST). Herein, we demonstrate the ability to use ϕA1122::*luxAB* to determine minimal inhibitory concentration (MIC) values and antibiotic susceptibility categories for various *Y. pestis* therapeutic antibiotics. We confirmed the assay by testing several nonvirulent *Y. pestis* isolates with reduced susceptibility to doxycycline or ciprofloxacin. Moreover, the assay can be performed directly on positive human blood cultures. Furthermore, as *Y. pestis* may naturally or deliberately be spread in the environment, we demonstrate the compatibility of this direct method for this scenario. This direct phage-based ID-AST shortens the time needed for standard AST to less than a day, enabling rapid and correct treatment, which may also prevent the spread of the disease.

## 1. Introduction

*Yersinia pestis*, the causative agent of plague disease, is a highly lethal pathogen [1] that impacted the history of humankind through three worldwide pandemics [2,3]. Based on its rapid disease progression, lethality and person-to-person transmission, *Y. pestis* is recognized by the Centers for Disease Control and Prevention (CDC) as a Tier-1 select agent. Plague is currently recognized as a re-emerging disease, as it is still present in Asia, Africa and America [2]. The last large outbreak of plague took place in Madagascar in 2017, where many of the patients conducted pneumonic plague [4]. Pneumonic plague is a severe form of the disease, demonstrating 100% mortality in untreated patients [5]. Moreover, it can cause death even in properly antibiotic-treated patients [6]. A recently report describing the death rate of probable or confirmed pneumonic plaque in the 2017 plague outbreak that occurred in Madagascar was 8–25% [7].

The early symptoms of plague are general nonspecific flu-like symptoms [1], resulting in a low index of suspicion among clinicians [8]. Pneumonic plague is an infectious disease and therefore has a high risk of causing a pandemic [9]. As shown in the recent COVID-19 pandemic, early detection of infected persons is a key factor for the isolation of transmittable patients and thus for rapid containment of the outbreak [10]. Additionally, rapid identification of the infectious agent allows proper treatment. In the case of plague disease, several antibiotics are the first choice for treatment, demonstrating high efficiency towards eliminating *Y. pestis* [11]. However, although most *Y. pestis* strains are susceptible to the recommended antibiotics, resistant strains have been described [12,13,14], demonstrating the need for conducting antibiotic susceptibility tests (ASTs) to identify the correct antibiotic treatment. Because a life-saving antibiotic treatment for the plague is only effective when initiated early after infection [5,15], antibiotic susceptibility testing of isolated *Y. pestis* must be performed rapidly.

The standard antibiotic susceptibility testing of bacteria includes a preliminary isolation step followed by an enrichment step [16]. The gold-standard AST method for *Y. pestis* is the broth-microdilution test, which lasts 24–48 h and must be initiated from isolated colonies on agar plates [16]. Overall, the process of detection and AST of *Y. pestis* may extend for a minimum of 3 days for positive blood culture (not including the incubation time needed for the culture to become positive) or a minimum of 5 days for *Y. pestis* isolated from environmental samples [17]. Since life-saving antibiotic treatment must be administered as close as possible to the onset of symptoms [5,15], the CDC recommends starting with drug therapy after laboratory specimens are collected. Rapid detection and AST methods may allow fast containment of infectious patients, as well as allow for the proper treatment of the disease [1]. Various rapid tests have been described for the detection and AST of *Y. pestis,* including immunoassays (detection [18,19] and AST [20]), genetic assays (detection [21] and AST [17,22,23]), optical assays (AST [24]) and bacteriophage-based assays [25].

Bacteriophages (phages) are viruses that specifically lyse and kill bacteria and can thus serve for specific bacterial detection assays [25,26]. Phage-based detection methods have the advantage of being simple and inexpensive to manufacture and store [25], preventing reagent shortages in cases of global need. Various phage-based detection methods for different pathogens have been developed based on native phages, phage peptides, biosensors, combinations of phages with PCR or immunoassays, and genetically engineered reporter phages (see recent reviews [25,27,28]).

A reporter-phage-based method for the detection of *Y. pestis* was developed by Schofield et al., who genetically engineered *Y. pestis*-specific lytic phage ϕA1122 to encode the luminescence gene cassette *luxAB* [29]. Because phages are dependent on bacterial systems, *luxAB* gene cassette is expressed only when a successful phage infection occurs [29], and following the addition of the n-decanal substrate solution, a bioluminescent signal can be monitored. This allows for the detection of viable bacteria only [26,27], as well as an evaluation of the bacterial concentration, seeing as the bioluminescent signal is correlated with the amount of infected bacteria. The identification of bacterial viability enables usage in reporter phages not only for ID but also for AST by detecting bacterial growth inhibition by the tested antibiotics [30].

In the present work, we expand the described usage of the ϕA1122::*luxAB* reporter phage for the testing of additional *Y. pestis*-recommended antibiotics and for other bacterial origins, such as positive human blood culture and environmental cultures spiked with *Y. pestis*. In addition, we optimized the assay incubation time to allow correct minimal inhibitory concentration (MIC) value determination using standard microdilution-derived MIC values as a reference. The optimized assay was confirmed by using several nonvirulent *Y. pestis* isolates with reduced susceptibility to doxycycline or ciprofloxacin.

## 2. Materials and Methods

### 2.1. Bacterial Strains

Experiments in this work were conducted using the nonvirulent *Y. pestis* strains Kimberley53Δ70Δ10 and EV76 [31]. Kimberley53Δ70Δ10 is resistant to streptomycin. Kimberley53Δ70Δ10 spontaneous mutants with reduced susceptibility to doxycycline [22] or to ciprofloxacin [17] were used for assessment of the phage-based AST and are listed in Table 1.

### 2.2. Bacteriophages

In this study, we used the reporter bacteriophage ϕA1122::*luxAB*, in which a luminescent gene cassette, *luxAB* (derived from *Photorohabdus luminescens* [32]), was inserted into ϕA1122 WT phage (kindly provided by Prof. Mikael Skurnik [33]. The *luxAB* cassette was inserted downstream of A1-A2-A3 *E. coli* early gene promoters, replacing 6 nucleotides (898–903) in the ϕA1122 phage genome, as previously described by Schofield et al. [29]. Bacteriophage stock was prepared by the agar overlay method [34]. Phage titration was performed by serial 10-fold dilutions in SM buffer (0.1 M NaCl, 8 mM MgSO_4_, 50 mM Tris-HCl pH 7.5 and 0.01% gelatin solution), followed by spot assay or plaque assay [31]. The phage stock was stored at 4 °C in the dark until use.

### 2.3. Growth Media, Buffers, Antibiotics and Human Whole Blood

All media powders were purchased from Bactlab Diagnostics Ltd. (Ceasarea, Israel) This includes brain–heart infusion broth (BHI; Cat. no. 237500), brain–heart infusion agar (BHIA; Cat. no. 241830), Bacto agar (Agar; Cat. no. 214010) and Muelle–Hinton broth (MHB; Cat. no. 212322). For top agar, 0.6% Bacto agar was added to BHI broth.

PBS (Dulbecco’s phosphate buffered saline, no calcium and no magnesium) was purchased from Biological Industries (Cat. no. 02-023-1A).

The LuxAB substrate solution n-decanal was purchased from SIGMA (St. Louis, MO, USA, Cat. no. D7384-25G) at a concentration of ≥98%. Prior to each experiment, a fresh 5% solution was made by dilution in PBS (1:20).

The antibiotic powders used in this study were chloramphenicol (CL, Sigma, C0378), streptomycin (SM, Sigma, S6501), gentamicin (GM, Sigma, G1264), doxycycline (DC, Sigma, D9891) and ciprofloxacin (CIP, Teva, CIPRO-TEVA 2 mg/mL). All antibiotics were suspended in water, and dilutions were performed according to the CLSI guidelines [16].

Human whole-blood donations (using CPDA1 as an anticoagulant) were provided by the Israeli Blood Bank and were kept at 4 °C until use. The blood samples were used within 2 weeks of collection.

### 2.4. Preparation of Blood-Culture-Derived Y. pestis

Isolated *Y. pestis* colonies (grown on BHIA plates at 28 °C for 48 h) were suspended in BHI to an optical density (OD)_660nm_ value of 0.35 (~1–3 × 10^8^ CFU/mL), and dilution was performed as required. The bacterial suspension was then diluted 1:100 with whole human blood (10 mL) and injected into an aerobic blood culture bottle (BACTEC Plus Aerobic/F Culture Vials; BD, Cat. no. 442192). The bottle was incubated in a BACTEC™ FX40 instrument until it was indicated positive by this system. For the isolation of *Y. pestis* bacteria from blood components, we used the serum separation tube (SST) separation protocol as we described previously [35]. Briefly, 6 mL of blood culture was injected into the SST (Grainer Bio-One, Cat. no. 455071) and centrifuged at 1700× *g* for 15 min at room temperature (RT). The supernatant was discarded, and the bacterial lining on the gel matrix was resuspended to the original volume using MHB growth media. Resuspended bacteria were used for diagnostic tests, including identifying the bacteria by the specific phage-based bioluminescent assay and simultaneously determining their antibiotic susceptibility to various antibiotic agents by phage-based AST.

### 2.5. Preparation of Y. pestis-Spiked Environmental Samples

Environmental samples (asphalt) were collected by wiping a square of 20 × 20 cm^2^ using three cotton applicators predipped in PBS (sterile wooden applicator cotton tipped individually wrapped; Copan, Murrieta, CA, USA, Cat. no. 165KS01). Five milliliters of PBS were added to a 50 mL conical tube containing the three applicators and vortexed well. Applicators were pressed against the tube wall to extract residual liquid before removal. The tube was left to stand for 2 min at RT to allow large dirt particles to sink, and the supernatant was transferred to a clean tube. For spiking *Y. pestis* bacteria into the environmental sample, isolated colonies (grown on BHIA plates at 28° C for 48 h) were suspended in PBS to an OD_660nm_ value of 0.35 (~1–3 × 10^8^ CFU/mL), and dilution was performed as required. The PBS-suspended *Y. pestis* (1 mL) was transferred into 4 mL of the environment supernatant solution, and the spiked sample (hereinafter “environmental sample”) was diluted 1:3 with MHB growth medium. The environmental sample was divided into two parts: one part (5 mL) was immediately tested to detect *Y. pestis* in the sample via the luminescence assay (as described below), and the other part (10 mL) was simultaneously used to determine the bacterial antibiotic susceptibility by phage-based AST. For phage-based AST, the environmental sample was incubated at 28 °C at 200 rpm for 1 h for MHB adjustment. After 1 h, the sample was diluted (if needed) to ~10^6^ CFU/mL based on the bioluminescent signal obtained in the bioluminescence assay (10-fold serial dilutions to obtain relative luminescent units (RLUs) = 100–500) and tested by phage-dependent AST.

### 2.6. Detection of Y. pestis by Reporter-Phage-Based Luminescence Assay

A blood-culture- or environment-derived suspension was tested for *Y. pestis* identification by detecting the bioluminescent signal following infection with the ϕA1122::*luxAB* reporter phage. Two hundred microliters of the bacterial sample was inoculated into 3 wells of a 96-well white microplate (Thermo Scientific Nunc, Rochester, NY, USA, Cat. no. 165306), and 20 µL of the reporter phage ϕA1122::*luxAB* (2.2 × 10^9^ PFU/mL) was added to each well (addition of phage represents Time = 0). The microplate was incubated in a SPARK 10M plate reader (TECAN, Salzburg, Austria) at 28 °C, and bioluminescence was determined at 20, 40 and 60 min after phage addition. Prior to each read, 20 µL of 5% n-decanal LuxAB substrate solution was injected into the relevant wells by the plate readers’ injector followed by microplate shaking for 5 s and bioluminescent signal reading for 10 s. Each well was read one time only. Significant positive signal in the test was determined as RLU ≥ 100 (S/N ≥ 2.5), following the calculation of the average of three triplicate wells after deleting background luminescence (RLU of wells without added n-decanal). The noise value (average background value) was determined for each experiment.

### 2.7. Standard Microdilution Susceptibility Test

A standard microdilution test was performed according to the CLSI guidelines [16] using a 96-well transparent microplate (Techno Plastic products, Trasadingen, Switzerland, Cat. no. 92696). Antibiotics were serially diluted twofold with MHB to a concentration range that included the microdilution MIC value and above and below this concentration, using twice the final concentration required in the well (final concentration ranges are displayed in Table 2), and 50 µL of the various antibiotic solutions was added to the microplate wells. Prepared microplates were kept at −70 °C until use and were defrosted to RT prior to use. Bacteria were inoculated on BHIA plates at 28 °C for 48 h, suspended in MHB to OD_660nm_ = 0.35 (~10^8^ CFU/mL) and diluted 1:100 with MHB. Fifty microliters of diluted bacteria was added to each well of the prepared microplate. The microplate was incubated at 28 °C for 24 h in a plate reader (SPARK 10M, TECAN, Salzburg, Austria), and the optical density at 630 nm (OD_630_) was measured at the endpoint. OD_630_ values represent the average values obtained for 3 wells of bacteria inoculated in triplicate after subtracting the average blank (MHB only) values. The MIC value was defined after 24 h of growth as the lowest antibiotic concentration that reduced bacterial growth to less than 10% of the growth control with no added antibiotic. No growth was verified by unaided visual inspection.

### 2.8. Reporter-Phage-Based AST

For reporter-phage-based AST, 96-well white microplates (Thermo Scientific Nunc, Rochester, NY, USA, Cat. no. 165306) were prepared in advance, as described for the standard microdilution test, with the difference of the administration of 100 µL of the MHB diluted antibiotic solutions. The ranges of antibiotic concentrations were as described for the standard microdilution test. Microplates were kept at −70 °C and defrosted to RT prior to use. One hundred microliters of bacterial suspension (prepared either as described for standard microdilution or as described for environment/blood culture samples) was added to each well, and the microplate was incubated for several hours at 28 °C, as stated in each figure’s legend. After the antibiotic exposure period, 20 µL of ϕA1122::*luxAB* reporter phage solution (2.2 × 10^9^ PFU/mL) was added to each well. The microplate was incubated in a SPARK 10M plate reader at 28 °C, and bioluminescence was assessed 30 min after phage administration, as described above for the bioluminescence assay (n-decanal injection, shaking and 10 s read). The MIC values were determined as the minimal antibiotic concentration that showed a bioluminescence value less than 10% of the growth control bioluminescence obtained value (RLU values after removing background value, as described above). The results are the average of triplicate wells, and error bars represent the STDEV.

## 3. Results

### 3.1. Rapid Detection of Y. pestis Using a Bioluminescent Reporter Phage

**Figure 1:**
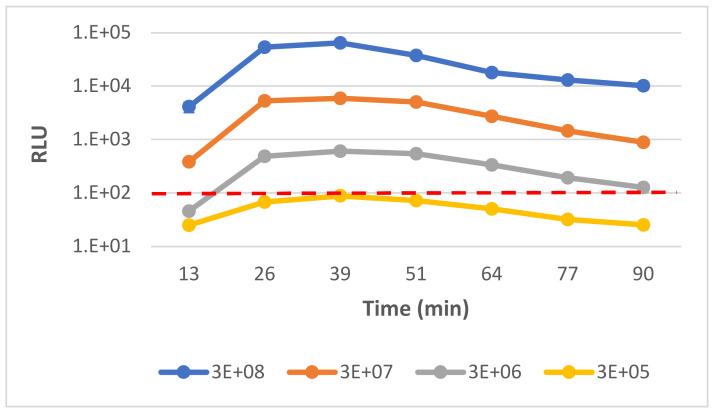
Determining the limit of detection of *Y. pestis* phage-based diagnostic assay. Detection of *Y. pestis* Kimberley53Δ70Δ10 strain was tested by phage-based assay. BHI-suspended bacteria were diluted 1:10 with BHI growth medium to a concentration range of 3 × 10^8^–3 × 10^5^ CFU/mL. Diluted cultures were added to a 96-well plate (0.2 mL/well) followed by ϕA1122::*luxAB* administration (20 µL/well) and incubation at 28 °C in a SPARK 10M plate reader. The bioluminescent signal (RLU) was detected, following 5% n-decanal substrate injection (20 µL/well). The results are representative of one out of three independent biological replicates. Values are the mean of results obtained in three triplicate wells in one biological experiment (error bars represent the standard deviation/STDEV (in some cases error bars are smaller than the symbol size). The limit of detection (RLU = 100) is marked by a red dashed line.

To rapidly identify and determine the antibiotic susceptibility of *Y. pestis* bacteria, we expanded the previously published usage of the ϕA1122::*luxAB* reporter phage for *Y. pestis* identification in blood- and environment-derived cultures [29]. We determined the assay’s limit of detection (LOD) in BHI-suspended *Y. pestis* culture. A positive luminescent signal was defined as 100 relative luminescent unit/RLU (2.5 × background luminescent signal of growth media only). As shown in Figure 1, the phage-based bioluminescent signal was correlated with the bacterial concentration, and the limit of detection (in which RLU ≥ 100) was 1 × 10^6^ CFU/mL. The test is rapid, and a positive signal arose within 10–30 min after phage administration, depending on the culture concentration. Thus, we further applied the reporter phage for the rapid AST.

### 3.2. Optimization of the Exposure Period to Antibiotics for the Reporter-Phage-Based AST

Determining bacterial susceptibility to antibiotics was performed by testing the ratio of bacterial growth in the presence of serial twofold diluted antibiotic to a growth control with no added antibiotic. The observed correlation between the reporter-phage-derived bioluminescent signals (RLU) and bacterial concentrations (Figure 1) can allow for the assessment of bacterial growth in the presence of antibiotics in comparison to bacterial growth in antibiotic-free media by monitoring the phage-derived bioluminescent signal. For the development of phage-based AST, we relied on the CLSI guidelines required for standard broth-microdilution AST [16]. This includes the usage of a standard bacterial inoculum of 5 × 10^5^ CFU/mL, the usage of Mueller–Hinton (MH) growth medium and culture exposure to serial twofold dilutions of the tested antibiotics in the relevant concentration range. Phage-based AST consists of four steps: (I) the exposure of bacterial culture to various concentrations of the tested antibiotics; (II) infection with the reporter phage ϕA1122::*luxAB* and incubation for 30 min; (III) the administration of the n-decanal substrate and the analysis of the bioluminescent signal obtained in antibiotic-exposed cultures compared to the growth control culture; and (IV) MIC value determination. As in the standard microdilution test, where the MIC value is determined as the minimal antibiotic concentration leading to growth inhibition (observed by the unaided eye [16] or by ≥90% reduction in OD measurement [17]), we determined the MIC value obtained in the phage-based AST as the minimal antibiotic concentration that showed a bioluminescent signal lower than 10% of the bioluminescent signal of the growth control.

**Figure 2:**
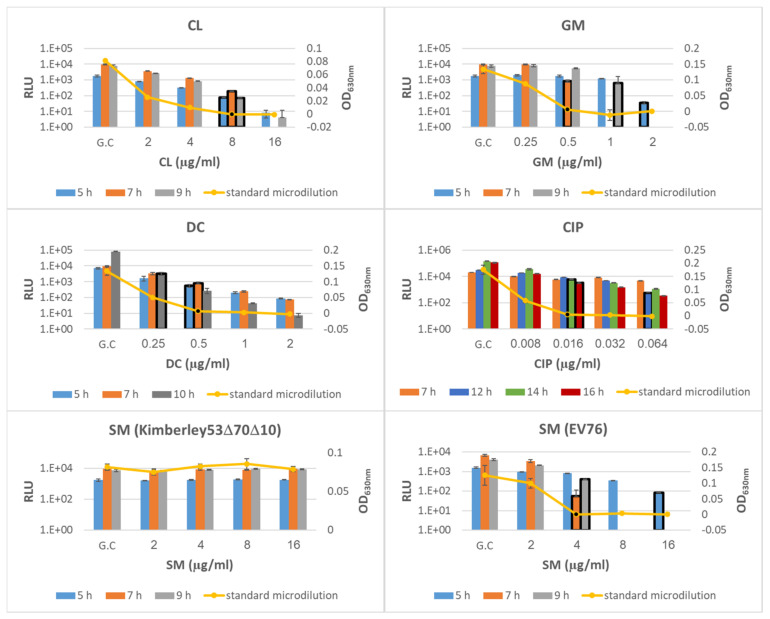
Optimization of the antibiotic exposure period for phage-based AST. *Y. pestis* strains Kimberley53Δ70Δ10 and EV76 were suspended in MHB to a concentration of ~5 × 10^5^ CFU/mL and were tested simultaneously by phage-based and standard microdilution AST. Bacterial suspensions were divided into 96-well microplates (50 µL/well) already containing 50 µL of each of 5 different antibiotics—CL (chloramphenicol), SM (streptomycin), GM (gentamycin), DC (doxycycline), and CIP (ciprofloxacin)—at various concentration ranges (detailed in Table 2); the microplates were then incubated at 28 °C. For phage-based AST, exposure periods were as follows: CL, SM and GM were tested at 5, 7 and 9 h of exposure; DC was tested at 5, 7 and 10 h; and CIP was tested at 7, 12, 14 and 16 h. All antibiotics were tested using Kimberley53Δ70Δ10, and SM was also tested using EV76. Each graph displays the resulting bioluminescent signals (RLU; bars) of all exposure times and the OD_630_ values obtained in the standard microdilution tests (yellow curve) following 24 h of antibiotic exposure using one bacterial strain exposed to one antibiotic. MIC value obtained in the phage-based AST was determines as the minimal antibiotic concentration that showed a bioluminescent signal lower than 10% of the bioluminescent signal of the growth control. The MIC values obtained by phage-based AST are marked by a black frame around the relevant bar, and the MIC values obtained by the microdilution test are marked by a black dot. Values are the mean results of 3 wells in the same experiment (error bars represent the standard deviation). GC = Growth Control.

To determine the minimal antibiotic exposure time required for reliable phage-based AST, we exposed *Y. pestis* Kimberley53Δ70Δ10 culture to the tested antibiotics for several time periods and compared the resulting MIC values of each exposure time to the MIC values of the standard microdilution test (obtained following 24 h of antibiotic exposure). As Kimberley53Δ70Δ10 is resistant to streptomycin (SM), this antibiotic was tested using the SM-sensitive *Y. pestis* EV76 strain. As shown in Figure 2, out of the three tested exposure times (5, 7 and 9/10 h), accurate MIC values were obtained at 7 h of antibiotic exposure for CL, SM, GM and DC (*Y. pestis* MIC interpretive criteria for all 5 antibiotics are listed in Table 2). Nevertheless, the phage-mediated MIC value obtained for CIP was not accurate even after 7 h of antibiotic exposure, which led us to prolong the antibiotic exposure periods, as shown in Figure 2. Longer exposure times of 14 or 16 h resulted in an accurate MIC value for CIP. Thus, we defined an optimal exposure time of 7 h for CL, SM, GM and DC and 14 h for CIP. The application of the phage-based AST for the *Y. pestis* EV76 strain indicated that the assay may be universally applied to additional *Y. pestis* strains, as all phage-based MIC values were identical to the standard values (Figure S1).

### 3.3. Phage-Based AST for Y. pestis Derivates with Reduced Susceptibility to DC or CIP

**Figure 3:**
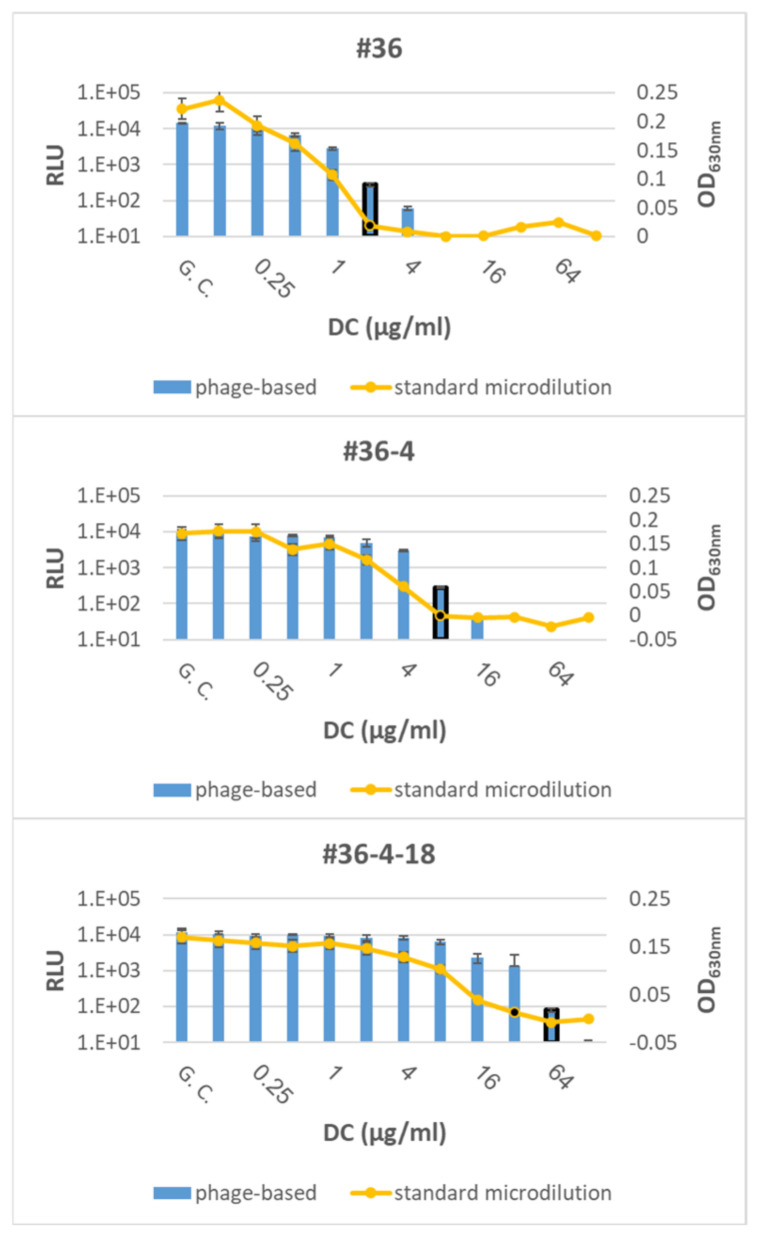
Phage-based AST for Kimberley53Δ70Δ10 derivatives with reduced susceptibility to doxycycline. Three Kimberley53Δ70Δ10 derivatives with reduced susceptibility to doxycycline (DC) (see Table 1) were suspended in MHB, diluted to ~5 × 10^5^ CFU/mL and tested by the phage-based AST at 7 h of doxycycline exposure (blue bars) and by the standard microdilution test (yellow curve). The MIC values obtained by the phage-based AST are marked by a black frame around the relevant bar, and the MIC values obtained by the microdilution test are marked by a black dot. Values are the mean of three triplicate wells in the same experiment (error bars represent the standard deviation). GC = Growth Control.

**Figure 4:**
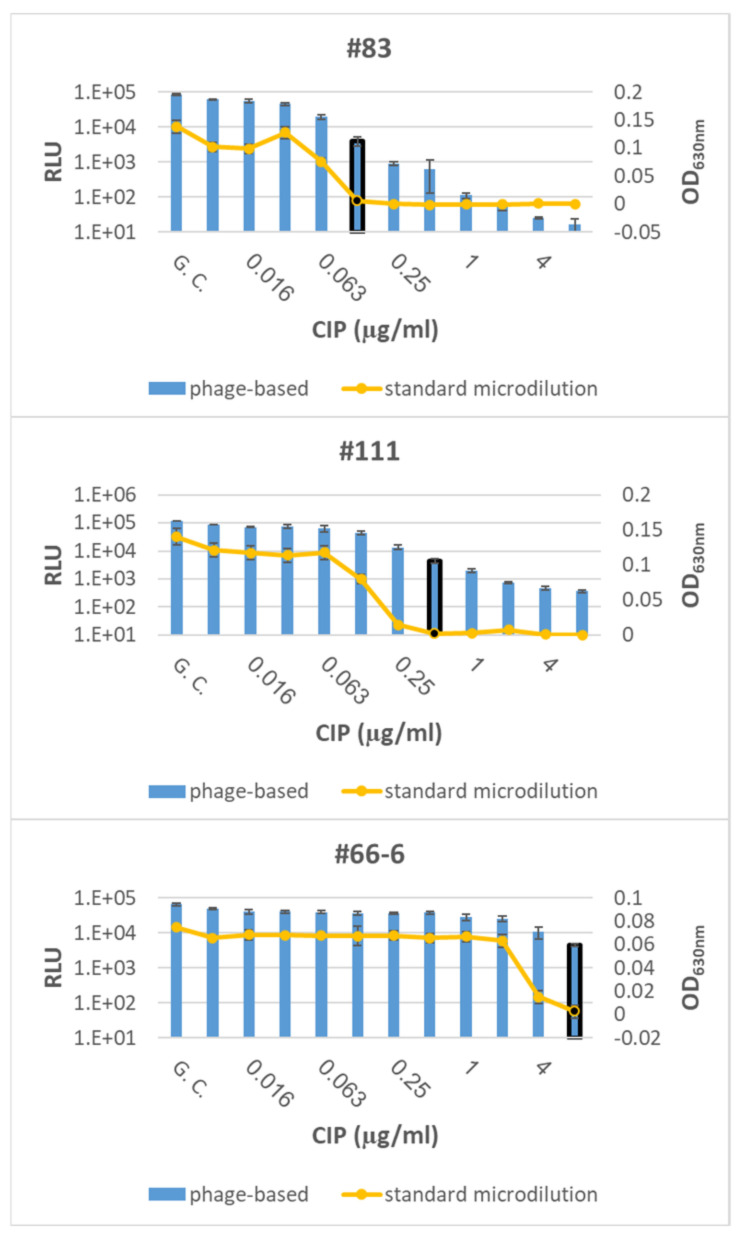
Phage-based AST for Kimberley53Δ70Δ10 derivatives with reduced susceptibility to ciprofloxacin. Three Kimberley53Δ70Δ10 derivatives with reduced susceptibility to ciprofloxacin (CIP) (Table 1) were suspended in MHB, diluted to ~5 × 10^5^ CFU/mL and tested by the phage-based AST at 14 h of ciprofloxacin exposure (blue bars) and by the standard microdilution test (yellow curve). The MIC values obtained by the phage-based AST are marked by a black frame around the relevant bar, and the MIC values obtained by the microdilution test are marked by a black dot. Values are the mean of three triplicate wells in the same experiment (error bars represent the standard deviation). GC = Growth Control.

The accuracy of the phage-based AST was assessed by testing *Y. pestis* Kimberley53Δ70Δ10 derivates with various MIC values (representing different susceptible categories; Table 2; [16]) for DC or CIP (Table 1). DC was selected as a representative of protein synthesis inhibiting antibiotics (required a 7 h exposure period), and CIP was selected as a representative of DNA replication inhibiting antibiotics (requires 14 h of antibiotic exposure). Both antibiotics are recommended by the CDC for plague treatment. As shown in Figure 3, two of the derivatives with reduced susceptibility to DC resulted in the same MIC values as the standard microdilution test, while the phage-mediated MIC of the third derivative (#36-4-18) was twofold higher than the standard microdilution MIC; however, there was no change in its susceptible category, which was defined as resistant. Moreover, all three derivatives with reduced susceptibility to CIP resulted in the same MIC value as that obtained in simultaneous microdilution AST (Figure 4).

### 3.4. Phage-Based Direct ID-AST of Y. pestis from Blood Cultures

To further shorten the duration of the phage-based ID-AST test, we analyzed *Y. pestis* spiked into and grown in human blood culture. We found that in the presence of blood, the detection of the luminescent signal was delayed, and a longer incubation time of 100 min was required for MIC determination (Figure S2A). We assumed that blood components block phage infection, as we recently published for the parent phage, ϕA1122 [31]. Therefore, we tested whether elimination of blood components would increase the phage-derived luminescent signal. We compared two different methods for bacterial isolation from blood components: differential centrifugation followed by collection of the plasma-suspended bacterial fraction (and discarding the blood cellular fraction) or elimination of all blood components (plasma and cellular fraction) by centrifugation in a serum separation tube (SST) followed by resuspension of the bacteria lining on the gel matrix in MHB growth medium. We found that although the SST method may cause some loss of bacteria (up to 50%), which was not observed in the differential centrifugation method, the luminescent signal derived following this isolation method was much higher (Figure S2A). The LOD was ~1 × 10^6^ CFU/mL, and the assay required a short incubation time of 40 min post phage administration, as was observed for BHIA-suspended *Y. pestis* (Figure S2B vs. Figure 1). Moreover, *Y. pestis* (Kimberley53Δ70Δ10 and EV76) spiked into and grown in human blood culture (incubated in a BACTEC™ FX40 device) until the culture turned positive, followed by SST isolation, could be identified within 40 min of phage infection (Figure S2C), which was not much longer than that observed for BHI-suspended bacteria at the same concentrations (Figure 1). Hence, the SST bacterial isolation method was selected for further development of the ID-AST assays.

**Figure 5:**
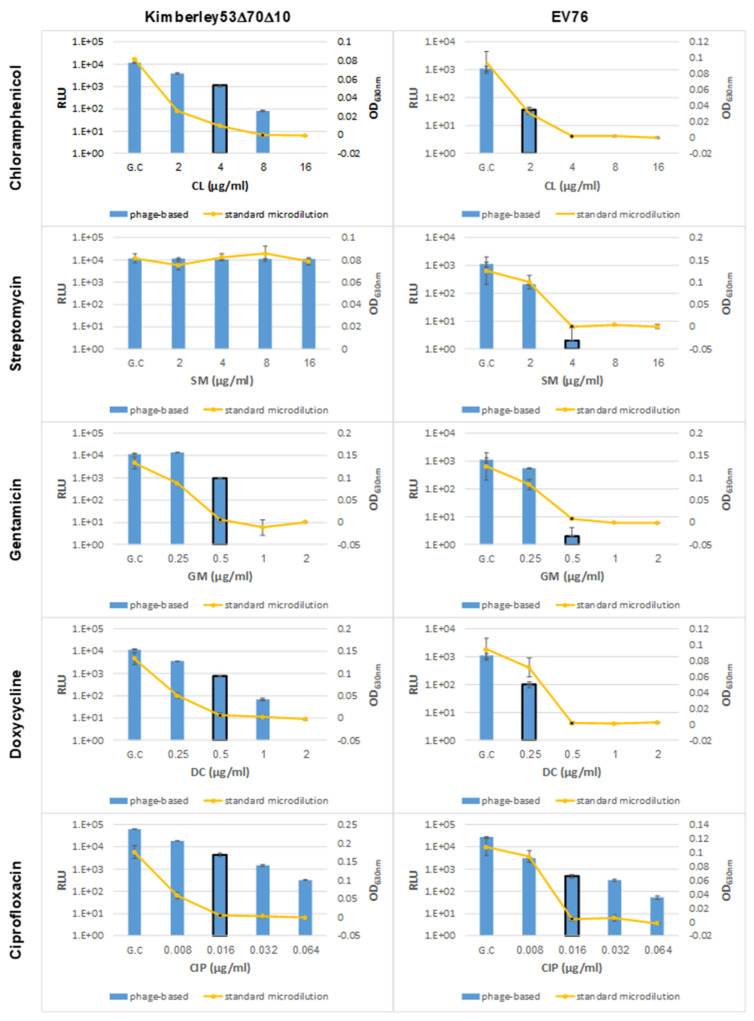
Direct phage-based AST of human blood cultures. *Y. pestis* strains Kimberley53Δ70Δ10 and EV76 were inoculated into human blood cultures in a Bactec aerobic plus/F bottle and incubated in a BACTEC FX40 instrument until a positive signal was obtained. Bacteria were separated from blood components by centrifugation of 8 mL of positive blood culture in an SST, resuspended in 8 mL of MHB and diluted 1:100 with MHB. The diluted culture was used for phage-based AST, with an exposure time of 7 h to CL, SM, GM and DC and 14 h to CIP. Bioluminescent values (RLU, blue bars) are displayed in comparison to OD_630_ values obtained by the standard microdilution test performed with antibiotic exposure of 5 × 10^5^ CFU/mL of MHB-suspended bacteria (yellow curves). The MIC values obtained by the phage-based AST are marked by a black frame around the relevant bar, and the MIC values obtained by the microdilution test are marked by a black dot. Values are the mean of three triplicate wells in the same experiment (error bars represent the standard deviation). GC = Growth Control.

Live counts of various *Y. pestis*-positive blood cultures incubated in the BACTEC™ FX40 instrument revealed that the bacterial concentrations at the time of alert were usually in the range of 1 × 10^7^–6 × 10^7^ CFU/mL [36]. Thus, as the required bacterial inoculum for a standard microdilution test is 5 × 10^5^–1 × 10^6^ CFU/mL [16], we diluted the SST-isolated bacteria 1:100 with MHB to achieve a proper bacterial concentration for the AST. The diluted samples were tested in phage-based AST for MIC value determination of five antibiotics at the optimized exposure periods of 14 h for CIP and 7 h for the other antibiotics. Two blood cultures of *Y. pestis* Kimberley53Δ70Δ10 and one blood culture of *Y. pestis* EV76 were tested for all five antibiotics. As shown in Figure 5, the phage-based MIC values were identical to the microdilution-derived MIC values, apart from the twofold reduction in the phage-based MIC value seen for CL (for both strains) and DC (for the EV76 strain). Thus, discarding blood components by the SST method enables direct ID-AST within a short period of 10–17 h.

### 3.5. Detection and AST of Y. pestis Directly from Environmental Asphalt Samples

Environmental asphalt samples were prepared by spiking *Y. pestis* into PBS suspended samples. We found that detection of *Y. pestis* directly from environmental samples using the bioluminescent reporter phage resulted in lower luminescent signal values (RLU) compared to RLU values detected in PBS-suspended homogenous culture using similar bacterial concentrations (Figure S3A; “Sample” vs. “Control”). When diluted 1:3 with MHB, the resulting RLU levels were similar to those obtained from equivalent concentrations of PBS-suspended homogenous bacteria. Diluted environmental samples were detectable within 20 min of phage infection, with an LOD of ~3 × 10^6^ CFU/mL in the original (prediluted) sample (Figure S3B). The threefold diluted environmental samples showed similar LOD values of ~1 × 10^6^ CFU/mL, as shown for homogenous BHI-suspended bacteria (see Figure S3B vs. Figure 1). RLU values were indicative of the bacterial concentration in the sample.

**Figure 6:**
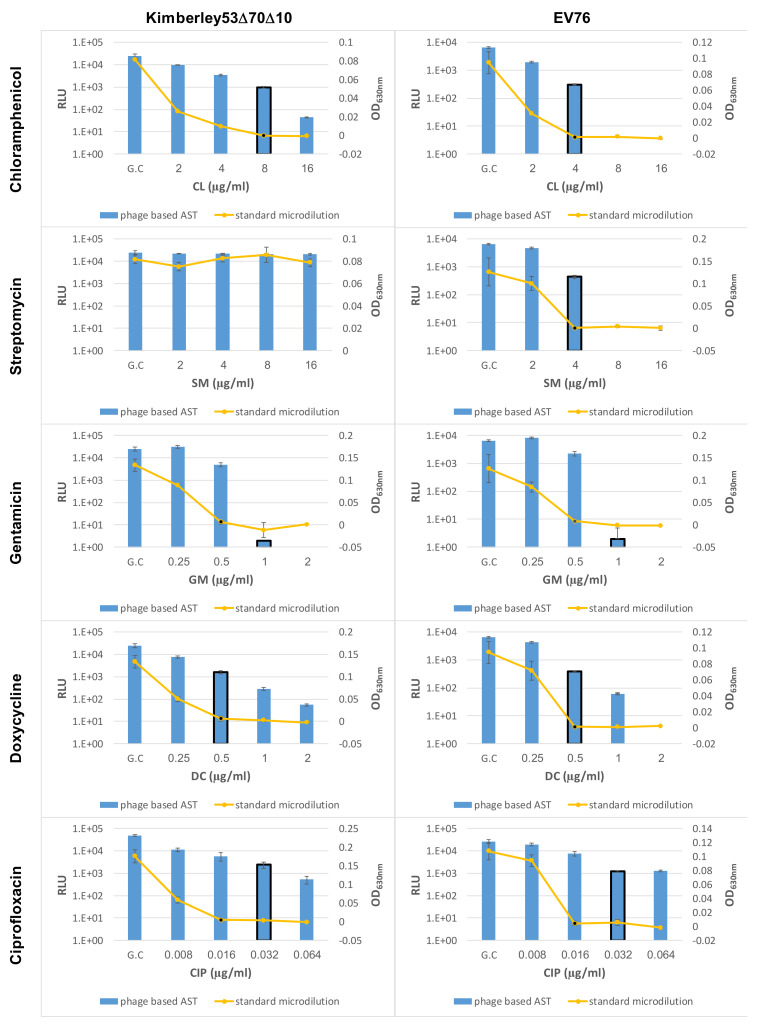
Direct phage-based AST of environmental samples spiked with *Y. pestis*. *Y. pestis* strains Kimberley53Δ70Δ10 or EV76 were inoculated into environmental asphalt samples prepared as described in the methods. Each inoculated culture was diluted 1:3 with MHB and tested by a phage-based bioluminescence ID assay, while the remaining sample was incubated at 28° C at 200 rpm for 1 h. After the evaluation of the bacterial concentration and dilution (if needed), the culture was tested by phage-based AST, with separate exposures to each of the five tested antibiotics, 7 h of exposure to CL, SM, GM and DC and 14 h of exposure to CIP. The resulting phage-based relative light units (RLUs, blue bars) are displayed in comparison to the OD_630_ values resulting from the standard microdilution test of MHB-suspended homogenous cultures (yellow curves). The MIC values of the phage-based AST are marked by a black frame around the relevant bar, and the MIC values obtained in the standard test are marked by a black dot. Values are the mean of three triplicate wells in one experiment (error bars represent the standard deviation). The experiment was performed in two independent biological duplicates for both *Y. pestis* strains, and the figure shows one representative experiment. GC = Growth Control.

Evaluation of the bacterial concentration pre-AST was performed by OD measurement of homogenous BHI-suspended bacteria. However, this method is not applicable for nonhomogeneous environmental samples. Since the RLU that is measured by the phage-based detection test is indicative of the bacterial concentration, the sample can be diluted to the desired concentration of ~10^6^ CFU/mL. In Figure 6, we show the phage-based AST results of *Y. pestis* spiked into environmental samples compared to the results obtained in the standard microdilution test using homogenous BHI-suspended bacteria. We tested the *Y. pestis* strains Kimberley53Δ70Δ10 and EV76. The obtained phage-based AST MIC values were identical or within one twofold difference with the MIC values from the standard microdilution test.

## 4. Discussion

Plague epidemics that occurred in the past have killed millions of people and are still of major concern in endemic countries. In addition, there is a global concern of deliberate spreading of *Y. pestis* by bioterrorists [37]. Since *Y. pestis*-infected persons may develop a severe disease with a high risk of death if not rapidly treated with proper antibiotics [11], rapid identification and antibiotic susceptibility determination is needed.

**Figure 7:**
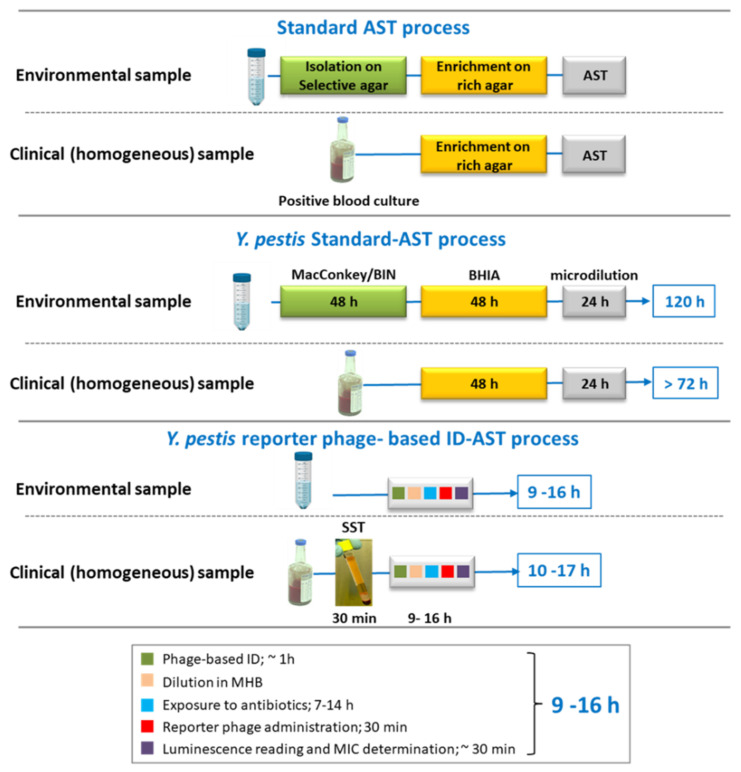
Standard or rapid *Y. pestis* phage-based ID-AST time scale.

To shorten the several days process of *Y. pestis* standard AST (Figure 7), various methods were developed. Those methods eliminate the pre-AST culturing step and/ or the AST step. For example, we have previously developed a rapid molecular AST, in which molecular alterations (quantifies by qRT-PCR) in the expression of specific mRNA markers, occurring soon after bacterial exposure to antibiotics, are translated to MIC values [17,22]. The molecular AST shortens the 24 h needed for the microdilution AST to 7 h assay (toward ciprofloxacin or doxycycline) [17,22]. Moreover, the molecular AST can be conducted directly on human blood culture derived *Y. pestis* with no need for the 48 h prior enrichment step [22]. However, as the gene expression profile is specific to the tested bacteria-antibiotic combination, a preliminary transcriptomic analysis and selection of proper mRNA markers should be performed for any additional antibiotic in question. In addition, specific primer sets for each tested antibiotic are required. Therefore, molecular AST, although rapid, is expensive and requires special devices and highly skilled laboratory employees, hence limiting its applicability in simple clinical laboratories or in point-of-care (POC) diagnostics. To overcome these limitations, we searched for a simple, inexpensive and rapid ID-AST method that can identify *Y. pestis* and follow its growth and viability directly from homogenous or heterogeneous culture, avoiding elongated isolation and/or enrichment steps. As bacteriophages are specifically targeted to their bacterial host, using a reporter phage enables ID-AST to be conducted without the need for a preliminary isolation and/or enrichment step.

Reporter-phage-based ID and AST assays were described previously for various pathogens (see recent review by Meile et al. [28]), including ϕA1122::*luxAB*, a *Y. pestis* specific reporter phage [38]. ϕA1122 lytic phage was chosen as a template for the reporter gene cassette insertion, since it is used by the CDC as a *Y. pestis* diagnostic tool (in a lysis test assay) and was shown to infect and lyse thousands of *Y. pestis* strains [30]. Universality was also described for the ϕA1122::*luxAB* reporter phage, as it was shown to identify 59 different *Y. pestis* virulent strains [38]. It should be mentioned that several non-*Y. pestis* strains also showed bioluminescent signals upon ϕA1122::*luxAB* infection but at a much lower level [38]. In addition to its described ability to rapidly identify *Y. pestis* strains, the potential of using the ϕA1122::*luxAB* reporter phage for rapid AST was described for either standard inoculum of *Y. pestis* suspended from agar plates [30] or human whole blood spiked with nonstandard (~100 CFU/mL) *Y. pestis* inoculum [38]. The ASTs were conducted for chloramphenicol, tetracycline and streptomycin exposure.

In the present article, we further adjusted the usage of the ϕA1122::*luxAB* reporter phage for rapid ID followed by AST toward additional antiplague recommended antibiotics, gentamycin, ciprofloxacin and doxycycline [11,39] and for additional relevant scenarios of both clinical and environmental settings. In addition, we optimized the assay to obtain correct MIC values and validated the assay by using *Y. pestis* derivates with various MIC values.

We constructed the ϕA1122::*luxAB* reporter phage and demonstrated its ability to identify *Y. pestis* strains by measuring bioluminescent signals upon phage administration. We conducted the diagnostic assay using different bacterial origins, including *Y. pestis* colonies suspended in rich growth media, spiked into and grown in human whole-blood culture (in Bactec aerobic^+^ bottles) or spiked into PBS-suspended environmental (asphalt) samples. It should be mentioned that to identify *Y. pestis* spiked into and grown in human blood culture, the removal of blood components prior to phage administration was needed as these components inhibit ϕA1122 infection [29,31]. Thus, we used the serum separation tubes (SSTs) for separating the cellular and plasma fractions from the bacteria. Further simplification of the assay could be done by using a phage that can infect in the present of blood components [31].

We found that the ID assay can be performed directly from clinical or environmental samples without the need to conduct an enrichment step (of 48 h) prior to the ID test. The assay was rapid, and a positive bioluminescent signal (RLU > 100) arose within 20–40 min of phage administration, depending on the bacterial concentration and origin (Figure 1 and Figures S2C and S3B). The assay’s LOD for the homogenous or the positive blood culture derived *Y. pestis* was 1 × 10^6^ CFU/mL, and since the bacterial concentration of a positive blood culture is >10^7^ CFU/mL [36], there is no need for further incubation. Notably, sensitivity of the assay is lower than other published reporter-phage-based ID assays [28], including the ϕA1122::*luxAB*-based assay [29]. This may result from our decision to define a higher threshold of 100 RLU in order to reduce the possibility of false-positive results. Reduction of the threshold may be considered, if testing of various environmental samples further confirms the low background of this assay. In addition, insertion of the reporter gene under the regulation of stronger promoter may also increase the sensitivity of the assay [30].

Additionally, in contrast to well-established genetic-based ID assays, such as the PCR method or immune-based ID assays, the reporter-phage-based ID assay differentiates between live and dead bacteria, as only metabolically active live bacteria enable the expression of the phage-encoded reporter genes *luxAB*. This characteristic is of crucial importance, especially for environmental samples, as identifying intentionally spread or naturally present live pathogens is of major importance for public health responses.

As the bacterial inoculum influences the resulting MIC value, especially in broth-based AST [17], we conducted ASTs by using the CLSI-required bacterial inoculum of 5 × 10^5^–1 × 10^6^ CFU/mL suspended in the required standard MH medium and conducted the tests in *Y. pestis* at an optimal in vitro growth temperature of 28 °C. AST was performed by bacterial exposure to the tested antibiotics followed by ϕA1122::*luxAB* administration and bioluminescence measurement.

We found that to obtain correct MIC values for the protein synthesis inhibiting agents-chloramphenicol, streptomycin, gentamicin and doxycycline, 7 h of antibiotic exposure was needed (Figure 2). It should be mentioned that for doxycycline and chloramphenicol, correct MIC values were obtained after 5 h of antibiotic exposure. However, as we aimed to develop a unified AST assay, which would include exposing a culture in the same microplate and for the same incubation period, we preferred the 7 h antibiotic exposure period, which allowed us to obtain correct MIC values for the four tested antibiotics. In contrast to the short exposure time (7 h) needed for the protein synthesis inhibiting agents, ciprofloxacin, a fluoroquinolone that inhibits DNA replication, required 14 h of antibiotic exposure before the correct MIC value could be obtained (Figure 2). As protein synthesis is not the primary mechanism of inhibition, phages can further inject their DNA into bacteria, and the reporter gene *luxAB* is further expressed. However, as antibiotic exposure is prolonged, secondary effects such as protein synthesis inhibition occur, and the inhibition of reporter gene expression is observed. 

Correct MIC values were obtained for *Y. pestis* derivates with reduced susceptibility, representing different susceptibility categories (including S/I/R) toward doxycycline (Figure 3) or ciprofloxacin (including S/non-S; Figure 4), thus strengthening the validity of the phage-based assay. Moreover, the phage-based 7 h AST could identify the streptomycin resistance phenotype observed for the Kimberley53Δ70Δ10 strain, as no reduction in the bioluminescent signal was seen even after 9 h of antibiotic exposure (Figure 2). As antibiotic resistance can influence bacterial metabolism and growth rates, it can impact the antibiotic exposure period required for correct MIC determination by the phage-based AST. The assessment of the phage-based AST protocol for the reduced susceptibility isolates showed that the 7 h/14 h exposure time needed for the *Y. pestis* WT strains was also suitable for these isolates, therefore allowing a unified exposure time for all tested bacteria.

To summarize, the phage-based AST: **1**—is conducted according to the standard CLSI guidelines, using a standard bacterial inoculum of 5 × 10^5^ − 1 × 10^6^ CFU/mL and the standard required MH rich medium; **2**—shortened the 24 h needed for the standard microdilution test to 7 h/14 h for the phage-based AST; **3**—resulted in correct MIC values (within the range of 1–2 twofold dilution) and antibiotic susceptibility category determination; **4**—showed accuracy in various *Y. pestis* derivates representing different standard MIC values and susceptibility categories; **5**—is suitable for a direct assay conducted on SST isolated *Y. pestis* derived from positive human blood culture, with no need for an agar enrichment step prior the AST; and **6**—was adjusted for AST performance of environmental samples spiked with *Y. pestis*. The reporter phage can differentiate between the *Y. pestis* target bacteria and the broad bacterial contaminants that exist in the environmental samples, thus eliminating the required long isolation step performed prior to standard, culture-based AST.

Herein, we show a rapid, simple, efficient and direct ID-AST method specific for *Y. pestis*. The direct phage-based AST shortened the required 3 days for analysis of clinical samples and the 5 days needed for environmental samples to only 7 h/14 h assays (Figure 7).

It should be mentioned that the time required for the blood culture to become positive (20–30 h, depending on inoculated bacterial concentration, [36]), prolongs the overall time to answer. In the case of resistant *Y. pestis* infected patient, this incubation period may be too long, and the patient may die. However, the present reporter-phage-based ID-AST method may serve as an efficient tool to rapidly improve antibiotic therapy, thereby preventing further infections.

## Figures and Tables

**Table 1:** *Y. pestis* Kimberley53Δ70Δ10 derivates with reduced susceptibility to doxycycline/ciprofloxacin.

Derivate #	MIC ^1^ (µg/mL)	Susceptibility Category ^2^	Resistance Mechanism
Doxycycline			
WT	0.5	S	
36	2	S	unknown
36-4	8	I	
36-4-18	32	R	
Ciprofloxacin			
WT	0.016	S	
83	0.125	S	single mutation in *gyrA*
111	0.5	Non-S	
66-6	8	Non-S	

^1^ MIC values were determined by the standard microdilution test [16]. ^2^ Antibiotic endpoint concentrations for susceptible category determination for *Y. pestis* were determined according to the Clinical and Laboratory Standards Institute (CLSI) guidelines [16].

**Table 2:** Antibiotic concentrations and *Y. pestis* MIC interpretive criteria.

Antibiotic	Final Concentration Range (µg/mL)	*Y. pestis* MIC Interpretive Criteria^1^		
		Sensitive	Intermediate	Resistant
Chloramphenicol (CL)	2–16	≤8	16	≥32
Streptomycin (SM)	2–16	≤4	8	≥16
Gentamycin (GM)	0.25–2	≤4	8	≥16
Doxycycline (DC)	0.25–2	≤4	8	≥16
Ciprofloxacin (CIP)	0.008–0.064	≤0.25		

^1^ Antibiotic endpoint concentrations for susceptible category determination for *Y. pestis* were determined according to the CLSI guidelines [16].

## Data Availability

Not relevant.

## Supplementary Materials

The following are available online at https://www.mdpi.com/article/10.3390/microorganisms9061278/s1: Figure S1: Phage-based AST of MHB-suspended EV76 toward CL, GM, DC and CIP. Figure S2: Detection of *Y. pestis* directly from positive human blood culture. Figure S3: Detection of *Y. pestis* directly from environmental asphalt samples.
