# Supplementary material for: Reporter-Phage-Based Detection and Antibiotic Susceptibility Testing of Yersinia pestis for a Rapid Plague Outbreak Response
Source: Microorganisms. 2021 Jun 11;9(6):1278. doi: 10.3390/microorganisms9061278 (PMC8231171; doi:10.3390/microorganisms9061278)
Supplement: Supplementary file 1 [file microorganisms-09-01278-s001.zip › microorganisms-1245654-supplementary.pdf]

Supplementary

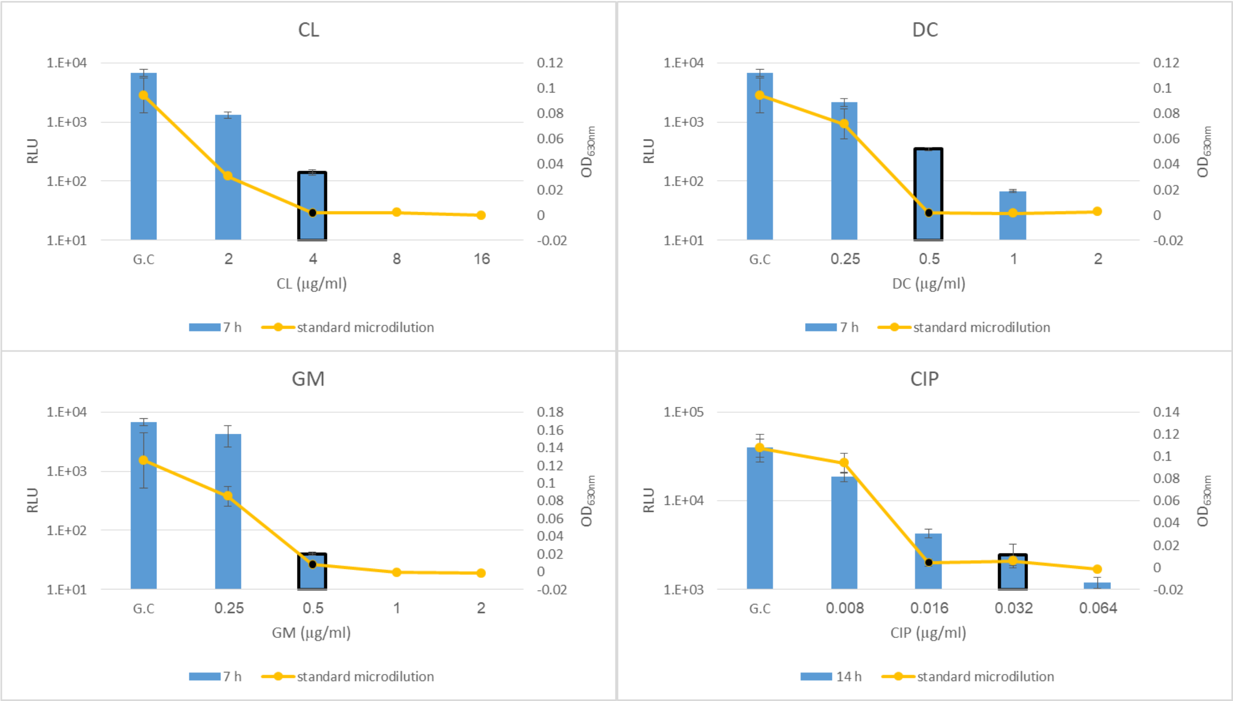

Figure S1. Phage-based AST of MHB-suspended EV76 toward CL, GM, DC and CIP. EV76 was tested by phage-based AST with 7 h of exposure to CL, GM and DC, and 14 h of exposure to CIP as described in the methods. Blue bars represent RLU values of phage-based AST (MIC marked by a black frame around the relevant bar) and mustard curves represent OD_{630nm} values of a standard microdilution test (MIC marked by a black dot) for comparison. The results are representative of one of three independent biological replicates. Values are the mean of 3 wells in one experiment (error bars represent standard deviation). G.C.= Growth Control.

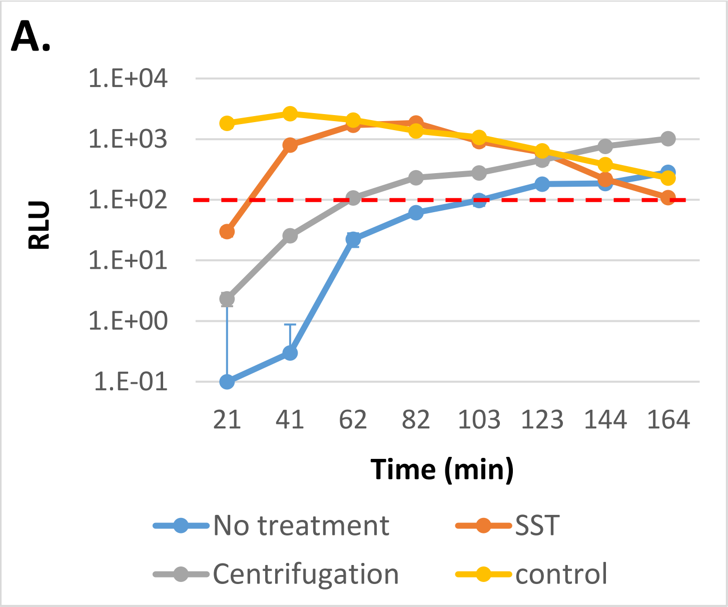

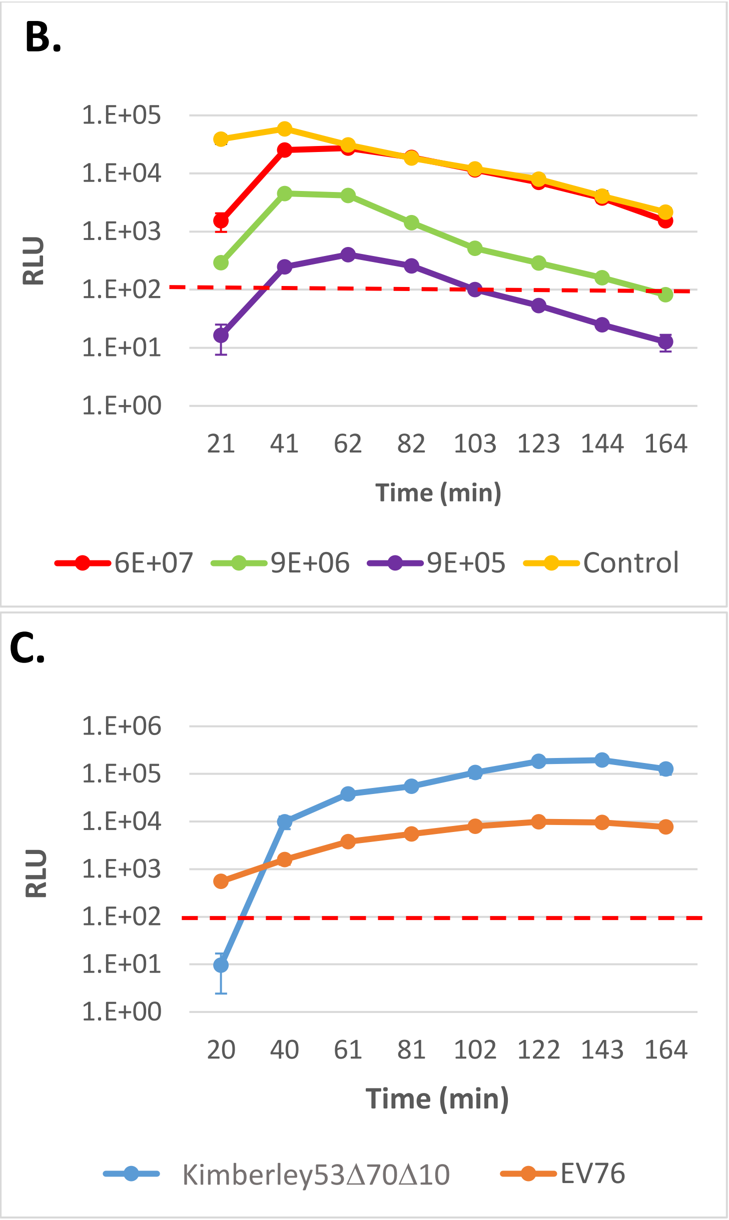

Figure S2. Detection of *Y. pestis* directly from positive human blood culture. **A.** *Y. pestis* strain Kimberley53Δ70Δ10 was inoculated (2×10^6 CFU/ml) into human blood cultures (10 ml human whole blood in Bactec aerobic plus/F bottle) and incubated at 180 × rpm, 37°C for 4 h (bacterial concentration at 4 h = 6.6×10^6 CFU/ml). Bacteria were separated from blood component by either centrifugation in serum separation tube (SST) or by differential centrifugation. SST separation was conducted as described at Methods section (bacterial concentration after SST = 6×10^6 CFU/ml). Differential centrifugation was done by centrifugation of 15 ml blood culture in a 50 ml tube at 300 × g, RT for 8 min, and the supernatant fraction containing the bacteria was transferred to a clean tube (bacterial concentration after differential centrifugation= 6.6×10^6 CFU/ml). For positive control, colonies of Kimberley53Δ70Δ10 grown on BHIA were resuspended to 2.2×10^7 CFU/ml. Phage-based ID assay was performed as described for Figure 1. **B.** For determining LOD of blood culture derived bacteria, 10-fold dilutions of Kimberley53Δ70Δ10 suspension were inoculated into three blood culture bottles and incubated for 4 h followed by SST purification and phage-based ID as described above. **C.** Human blood culture was spiked with either Kimberley53Δ70Δ10 (~10^4 CFU/ml) or EV76 (~10^4 CFU/ml) *Y. pestis* strains and incubated at the BACTEC FX40 devise until it became positive. Bacteria were purified by SST (bacterial concentration for Kimberley53Δ70Δ10 and EV76: 3×10^8 CFU/ml or 2.8×10^7 CFU/ml,

respectively) and ID assay was performed as described for A. Results are the average of 3 wells and error bars are STDEV (in some cases error bars are smaller than the symbol size).

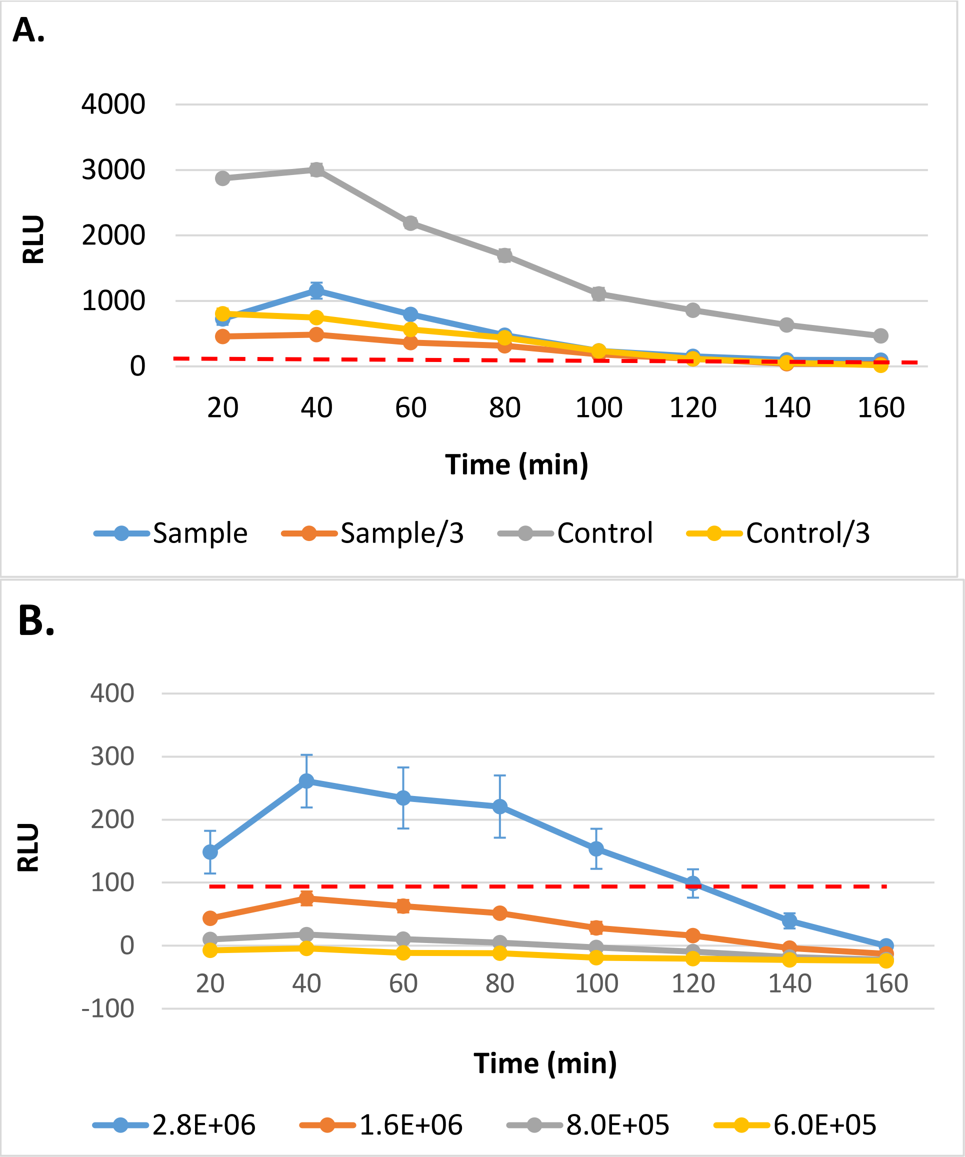

Figure S3. Detection of *Y. pestis* directly from environmental asphalt samples. A. Y. pestis Kimberley53Δ70Δ10 were resuspended in MH or in PBS and diluted to 2 × 10^6 CFU/ml. Half of each sample was diluted by 3 in MHB (to 6.6 × 10^5 CFU/ml). All 4 samples were tested by phage-based ID as described in the methods. B. Environmental samples were spiked with *Y. pestis* Kimberley53Δ70Δ10 in low concentrations, diluted by 3 in MH and tested by the detection method as described in the Method section. Numbers in the legend represent the bacterial concentration of each sample prior to dilution. Values are the mean of 3 triplicated in the same experiment, and the error bars represent the STDEV (in some cases error bars are smaller than the symbol size). The red dashed line represents the LOD.
